# Policymakers’ and other stakeholders’ perceptions of key considerations for health system decisions and the presentation of evidence to inform those considerations: an international survey

**DOI:** 10.1186/1478-4505-11-19

**Published:** 2013-05-24

**Authors:** Joshua P Vogel, Andrew D Oxman, Claire Glenton, Sarah Rosenbaum, Simon Lewin, A Metin Gülmezoglu, João Paulo Souza

**Affiliations:** 1 School of Population Health, Faculty of Medicine, Dentistry and Health Sciences, University of Western Australia, 35 Stirling Highway, Crawley 6009, Australia; 2 UNDP/UNFPA/UNICEF/WHO/World Bank Special Programme of Research, Development and Research Training in Human Reproduction (HRP) / Department of Reproductive Health and Research, World Health Organization, Avenue Appia 20, Geneva 1211, Switzerland; 3 Global Health Unit, Norwegian Knowledge Centre for the Health Services, P O Box 7004, St. Olavsplass, 0130, Oslo, Norway; 4 Health Systems Research Unit, Medical Research Council of South Africa, PO Box 19070, 7505, Tygerberg, South Africa

**Keywords:** Decide, Evidence summaries, Health system decisions

## Abstract

**Background:**

The DECIDE framework was developed to support evidence-informed health system decisions through evidence summaries tailored to health policymakers. The objective of this study was to determine policymakers’ perceptions regarding the criteria in the DECIDE framework and how best to summarise and present evidence to support health system decisions.

**Methods:**

We conducted an online survey of a diverse group of stakeholders with health system decision experience from 15 countries and the World Health Organization. We asked about perceptions of criteria relevant to making health system decisions, use of evidence, grading systems, and evidence summaries.

**Results:**

We received 112 responses (70% response rate). Most respondents had healthcare (85%) and research (79%) experience. They (99%) indicated that systematic consideration of the available evidence would help to improve health system decision-making processes and supported the use of evidence from other countries (94%) and grading systems (81%). All ten criteria in the DECIDE framework were rated as important in the decision-making process. Respondents had divergent views regarding whether the same (38%) or different (45%) grading systems should be used across different types of health decisions. All components of our evidence summary were rated as important by over 90% of respondents.

**Conclusions:**

Survey respondents were supportive of the DECIDE framework for health system decisions and the use of succinct summaries of the estimated size of effects and the quality of evidence. It is uncertain whether the findings of this survey represent the views of policymakers with little or no healthcare and research experience.

## Background

Decisions regarding health systems are often political processes involving a number of policymakers and other key stakeholders. There is a need to support these decisions with the best available evidence, however, the effects of health system interventions are often uncertain and stakeholders may have a low level of medical and research literacy. The Developing and Evaluating Communication Strategies to Support Informed Decisions and Practice Based on Evidence (DECIDE) project is a collaborative research project funded by the European Commission’s Seventh Framework Programme [http://www.decide-collaboration.eu]. The project’s objective is to develop and evaluate communication strategies to support evidence-informed decisions by building on the work of the Grading of Recommendations Assessment, Development, and Evaluation (GRADE) Working Group [http://www.gradeworkinggroup.org] and the Cochrane Applicability and Recommendations Methods Group [http://www.armg.cochrane.org]. As part of the DECIDE project, we have developed a framework for communicating evidence to inform decisions about health systems [http://www.decide-collaboration.eu/WP5/Strategies/Framework].

The development of this framework was informed by our earlier work on plain language summaries of systematic reviews [1] and work on evidence-based policy briefs [2-4]. The framework includes relevant criteria for making health system decisions as well as evidence to inform judgements about each criterion. The criteria included in the framework are seriousness of the problem, number of people affected, quality of the evidence, size of the benefits, size of the adverse effects, resource use (costs), value for money, impacts on equity, implementability (feasibility), and acceptability. These framework criteria emerged from a literature review, brainstorming, feedback from stakeholders, and application of the framework to examples. This survey, along with user testing and further application of the framework to health system decisions, will further inform the selection of criteria included in the framework and how they are presented.

There are several different systems available to grade the quality of evidence on the effects of healthcare interventions. Most of these have been used primarily for clinical practice guidelines and those systems have become increasingly similar to GRADE or replaced by GRADE [5-7]. Other systems have been used for population health guidelines (primarily for public health rather than health system interventions), such as the systems used by the Task Force on Community Preventive Services [6,8,9], although the GRADE system is also used for public health and health system recommendations [10,11]. All of these grading systems have focused primarily on grading the quality of evidence and only to a lesser extent, if at all, on frameworks for going from evidence to public health or health system decisions or recommendations.

The use of grading systems for rating the quality of evidence is seen as essential for evidence-based guideline development. The GRADE methodology has been adopted by WHO, as well as by national guideline developers and others to assess the quality of evidence in guideline development as it enables a comprehensive, transparent and structured analysis of available literature, whilst clearly communicating the quality of evidence and strength of the recommendations [1,12,13]. However, there is considerable debate as to whether the same approach to grading evidence for clinical decisions should also be used for health system decisions. Advocates for the use of the same grading system for both types of decisions state it can minimise confusion, reduce the risk of bias, and maintain transparency and consistency across different types of decisions. Critics state that a rating system that is appropriate for clinical interventions may not discriminate evidence in a way that is appropriate for health system programmes or policies, potentially disadvantaging effective interventions that are not amenable to randomized controlled trials [10].

The presentation of evidence to inform health system decisions requires the development of summaries that can 1) communicate complex information in plain language; 2) clearly present the anticipated effects and the certainty (quality) of the underlying evidence; and 3) effectively convey uncertainty. We have previously developed and user-tested evidence summaries tailored for health policymakers in low- and middle-income countries [2], built on earlier findings for Cochrane reviews [14,15]. An evidence summary based on the DECIDE framework needs to clearly communicate evidence relating to each of the ten DECIDE framework criteria, including the effect sizes and quality of evidence available for the anticipated desirable and undesirable effects of the intervention being considered. The design of these evidence summaries to support policymakers’ understanding of evidence therefore requires a detailed analysis of their perceptions of certainty (quality) of the evidence and how it is assessed.

To obtain a better understanding of policymakers’, managers’ and other stakeholders’ perceptions of the criteria for health system decisions in the DECIDE framework, and how evidence on these should be presented, we conducted an international online survey. The survey focused on stakeholders’ perceptions of each of the ten DECIDE framework criteria, the grading of health system evidence, and the use of evidence summaries. This paper aimed to collect information regarding the experience and perceptions of participants with respect to the proposed criteria, assessments of the quality of evidence used to inform judgements about the effects of interventions, and summaries of research evidence of the effects of health system interventions.

## Methods

We conducted an international online survey to determine perceptions of the importance and use of the criteria within the DECIDE framework and evidence summaries. We aimed to survey a diverse (rather than representative) group of policymakers from different countries, with a wide range of experience with different types of health policy and management decisions and with different perspectives. Our sampling frame included the nine DECIDE partner countries (Canada, England and Wales, Finland, Germany, Italy, the Netherlands, Norway, Scotland, and Spain), the World Health Organization (WHO), and six SURE (Supporting the Use of Research Evidence for policy in African health systems) partner countries, namely Cameroon, Ethiopia, Mozambique, Zambia, Uganda, and South Africa [http://www.who.int/evidence/sure/en/]. SURE is a related collaborative research project with the objective of developing and evaluating strategies for improving access to and use of research evidence in health policy development in Africa. These countries were included as we had sufficient networks linked to the DECIDE and SURE projects to provide the necessary referrals to potential participants. Inclusion criteria for survey participants included people responsible for health system decisions and stakeholders with an interest in and experience with health system decisions, such as delivery, governance and financial arrangements, and strategies for implementing health system changes. This could include public officials, managers, health workers, and representatives of special interest groups, international organizations, non-governmental organisations, donor countries, or the general public. Our partners in the DECIDE and SURE projects from each country and other personal contacts helped us to identify 10 to 20 people who were not involved with the DECIDE collaboration in each of the 15 countries and within WHO, with the aim of obtaining 10 completed questionnaires from each. We prepared an online survey (in English only) using the LimeService online platform [https://www.limeservice.com/] (Additional file 1 Table S1). The survey was revised after pilot testing in a small group of policymakers who provided feedback on the content, length, clarity, and ease of use. Informed consent was obtained from survey participants prior to commencing the survey and results were de-identified when exported for analysis to protect confidentiality.

To determine current use and perceived importance of the DECIDE criteria in health system decision-making, we asked participants to provide an example of a health system decision with which they had been involved or were familiar. Participants were asked to rate whether each of the ten criteria in the DECIDE framework (seriousness of the problem, number of people affected, quality of available evidence, benefits (desirable effects), adverse (undesirable) effects, resource use (cost), value for money, impacts on equity, implementability (feasibility), and acceptability) had been considered as part of this healthcare decision on a 3-point scale (yes, no, unsure). If a given criterion had not been considered, they were asked whether they considered it relevant, and also to state any other criteria they considered relevant. The opportunity to make optional comments was provided. Participants were also asked to rate the importance (important, probably important, not sure, probably not important, not important) of each of the ten criteria, with optional comments if desired.

To determine perceptions on the use of evidence in health system decisions, we asked participants about their use of 1) evidence from other countries; 2) systematic reviews to inform their health system decision; and 3) evidence grading systems. Specifically, we asked whether they believed that evidence grading systems should be the same or different for health system decisions, compared to clinical decisions. We also assessed the perceptions of participants as to the contents of summaries of evidence. These summaries, based on the best available evidence, should be concise yet include all of the key information needed to inform a health system decision. We asked participants to rate the importance of including certain types of information (components) in these summaries, namely: effect sizes, confidence intervals, numbers of studies, and the quality of the evidence. Participants also provided information on their research, healthcare, and decision-making experience.

Participants were contacted by email and asked to complete the online survey. Initial contacts were made by our partners in each country or directly by us. Non-responders received reminders via email at two and four weeks after the initial invitation. We summarised the results using frequencies and percentages, and collated provided comments. Our primary analysis focused on implications for our evidence to health system decisions framework and evidence summaries. We explored potential differences in responses across participants from different countries (DECIDE *versus* SURE partner countries) and across groups with different types of experience (with *versus* without research training or experience).

## Results

We received 112 responses (70% response rate) to the online survey. Of these, 84 responses (75%) were complete and 28 (25%) were partially complete. We received 23 responses (46% response rate) from the six SURE countries, 84 responses (93% response rate) from the nine DECIDE partner countries, and five responses (50% response rate) from WHO. Table 1 describes the characteristics of survey participants who provided background information (n = 84). Most (84.5%) of the respondents had healthcare professional training or had worked as a healthcare professional. The majority of these were physicians (87.1%), and most (69.0%) had over ten years clinical experience. Most (78.6%) also had some form of research training or experience; 54.5% of these had over ten years of research experience. Respondents had worked in a variety of organizations, most frequently in national governments (63.1%) and public organizations (48.8%). The most common forms of health system decisions made or supported by respondents were decisions regarding the selection of healthcare policies, reforms or programmes (77.4%), and decisions on their implementation (78.6%).

**Table 1:** Characteristics of survey participants

	**Survey participants, n (%)**
Total (completed entire survey)	84 (75.0)
No healthcare professional training or experience working as a healthcare professional	13 (15.5)
Healthcare professional training or experience working as healthcare professional	71 (84.5)
Physician	61 (85.9)
Nurse	1 (1.4)
Other	8 (11.3)
Missing	1 (1.4)
Years of experience as healthcare professional	
1 – 10 years	22 (31.0)
11 – 20 years	18 (25.4)
21 – 30 years	14 (19.7)
31 – 40 years	14 (19.7)
40+ years	3 (4.2)
Research training or experience working as a researcher	66 (78.6)
MSc or equivalent	24 (36.4)
PhD or equivalent	40 (60.6)
Other	2 (3.0)
Years of experience as a researcher	
1 – 10 years	30 (45.5)
11 – 20 years	20 (30.3)
21 – 30 years	12 (18.2)
31 – 40 years	3 (4.5)
40+ years	1 (1.5)
Levels of current and previous work^*^	
International governmental organization	15 (17.9)
National government	53 (63.1)
Regional government	26 (31.0)
Local government	18 (21.4)
Public organization	41 (48.8)
Private organization	16 (19.1)
Other	19 (22.6)
Types of health system decisions supported	
Experience representing the views of stakeholders in policy or management processes	46 (54.8)
Decisions about healthcare policies, reforms or programmes	65 (77.4)
Decisions about implementation of healthcare policies, reforms or programmes	66 (78.6)
Management decisions about health system arrangements	44 (52.4)
Decisions about quality improvement, patient safety or implementation of clinical guidelines	56 (66.7)
Other	5 (6.0)

^*^These categories were not mutually exclusive and hence the sum is greater than 100%.

**Figure 1:**
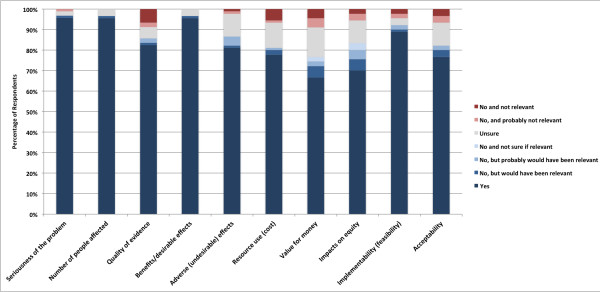
Survey responses regarding criteria considered for individual health system decisions.

We asked participants to describe a recent healthcare decision with which they had been involved or had the opportunity to follow closely. One hundred and two respondents (91.1%) had such an example and 10 (8.9%) respondents imagined a current or recent decision. Figure 1 shows their responses regarding whether our criteria were considered for their health system decisions. Over 75% of respondents stated they had considered these criteria in their decision, except for value for money (67%) and impacts on equity (70%). Comments were infrequent but almost universally in support of the need to consider these criteria and the lack of evidence on certain criteria, such as equity and implementation, for health system decisions. Two respondents indicated that consideration of these criteria is mandatory for decisions taken in their organization. Most (86.4%) of the respondents had considered evidence from other countries for their decision, and 94.3% of respondents agreed or somewhat agreed that evidence from other countries should be used to inform health system decisions. Most (90.9%) of the respondents stated they knew what a systematic review was. However, only 60.2% of respondents had used evidence from a systematic review to inform their decision. In response to our question on whether systematic consideration of the available research evidence would help to improve health system decision-making, 98.9% stated it would or it probably would.

**Figure 2:**
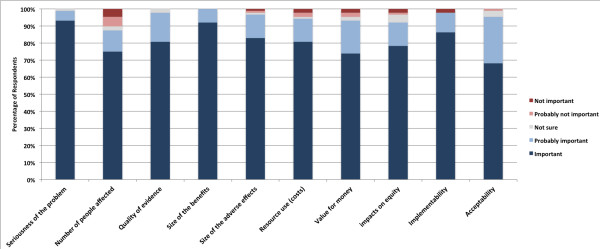
Survey responses rating the importance of criteria in health system decisions.

Respondent ratings on the importance of the ten criteria are summarised in Figure 2. Every criterion was rated as important or probably important by over 90% of respondents. Nearly all respondents (99.2%) agreed that explicit consideration of the ten criteria would help or probably help to improve health system decision-making. While comments supported the importance of the criteria, several respondents identified further criteria. These included sustainability of implemented changes, post-implementation monitoring systems, human resource implications, and “environmental” considerations (assessing what is occurring in nearby areas or similar jurisdictions).

**Figure 3:**
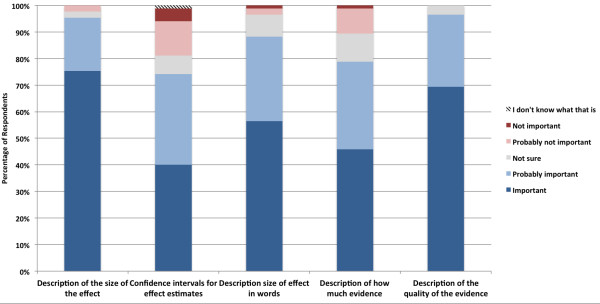
Survey responses rating the importance of components of a summary of evidence.

Most respondents agreed that a system of grading evidence would either definitely (59.7%) or probably (33.3%) improve health system decision-making processes (Table 2). When asked whether such grading systems should be the same for clinical and health system decisions, 38.4% said it should or probably should be the same, 45.3% indicated that different grading systems should or probably should be used for clinical and health system decisions, and 16.3% were neutral. This disagreement was also reflected in comments. Some respondents stated that using the same grading system improves consistency, transparency, and reproducibility, while others stated that they are fundamentally different types of decisions and evidence, requiring different grading systems. Two respondents (both from DECIDE partner countries) indicated an overall dislike of evidence grading systems. Respondent ratings on the importance of components of a summary of evidence are described in Figure 3. There was general agreement that all of the summary components were important or probably important, ranging from 79% (confidence intervals for effect estimates) to 96% (description of the quality of the evidence). All comments were in broad support, including several highlighting the need for clarity, simplicity, and brevity. Others suggested the use of disability-adjusted life years (DALYs) and number needed to treat (NNT) as important measures of effect.

**Table 2:** Respondents’ views on the use of grading systems to rate the quality of evidence of health decisions

	**Survey participants, n (%)**
**A system of grading the quality of evidence can help improve health system decision-making processes.**	
Yes	52 (59.7)
Probably	29 (33.3)
Not sure	5 (5.8)
Probably not	1 (1.2)
No	0 (0.0)
**Do you think that a system for rating the quality of evidence should be consistent for different types of decisions or that there should be different systems for different types of decisions (e.g., for clinical decisions and for health system decisions)?**	
The SAME SYSTEM DEFINITELY should be used for rating the quality of evidence for clinical and health system decisions	9 (10.5)
The SAME SYSTEM PROBABLY should be used for rating the quality of evidence for clinical and health system decisions	24 (27.9)
Neutral	14 (16.3)
A DIFFERENT SYSTEM PROBABLY should be used for rating the quality of evidence for health system decisions than for clinical decisions	27 (31.4)
A DIFFERENT SYSTEM DEFINITELY should be used for rating the quality of evidence for health system decisions than for clinical decisions	12 (13.9)

Overall, there was relatively little variation in the responses. Nonetheless, we explored potential differences in responses across participants from different countries and across groups with different types of research experience (Additional file 1 Table S1). We did not find any apparent differences in responses based on the respondents’ country (DECIDE *versus* SURE partner countries) or experience (with compared to without research training or experience).

## Discussion

We received 112 responses to an online survey from a diverse, international group of policymakers, managers, their support staff and other stakeholders to better understand their perceptions of the ten criteria in the DECIDE framework for going from evidence to health system decisions. We also obtained their perceptions of the components of summaries of evidence. Our respondents had a high level of professional healthcare and research experience and training and had worked in a wide range of levels and organizations. This may be due to the way in which they were identified, or a greater interest among those policymakers with clinical or research experience to participate in the survey. Our sample of policymakers was not intended to be representative of included countries, but was rather to obtain a diverse range of views from respondents.

Respondents had generally considered all ten criteria in the DECIDE framework in their own healthcare decisions and consistently rated these criteria as important or probably important to decision-making. In earlier work, Guindo and colleagues identified a range of healthcare decision criteria and criteria-based decision-making tools used in empirical studies of health system decisions – the most frequently cited criteria were equity and fairness, efficacy/effectiveness, stakeholder interests and pressures, cost effectiveness, strength of evidence, and safety [16]. In our survey, comments by respondents indicated that not only were similar criteria considered important to decision-making processes, but that in some organizations, their consideration is mandatory. However, only 60.2% reported using systematic reviews to inform decision making. This may reflect both a lack of systematic reviews addressing relevant questions, as well as other reasons for not using research evidence to inform health policy decisions, as reported elsewhere [17,18].

Most respondents agreed that a system of grading evidence would improve health system decisions; however, there was considerable disagreement as to whether these grading systems should be uniform across clinical and health system decisions. This is consistent with the ongoing debate in the literature on the application of the GRADE approach to a range of decisions [10,12]. While a common approach to grading evidence may reduce confusion, minimise conflicts of interest and enhance intellectual rigor, others have argued that this approach can be overly complex and can favour false negative conclusions. Additionally, interventions that are not amenable to randomised trials could be disadvantaged in terms of prioritisation, funding and implementation [12]. Criticisms made by our respondents of a uniform grading approach concur with the literature that there is a lack of available high-quality evidence on health system interventions. Two respondents also indicated an overall dislike of grading systems, citing a tendency to oversimplify complex issues or a lack of institutional capacity to provide training or support grading activities. However, the application of some form of systematic consideration of evidence to support health system decisions had broad support in our survey. Successful implementation of the GRADE approach for health system decision-making will need to address concerns about its applicability to health system evidence.

Effectively communicating complex information through summaries has been proven in the use of the GRADE summary of findings table [13,19] and SUPPORT summaries [2]. SUPPORT summaries summarise the best available evidence of the effects of health system interventions for low and middle-income countries. Such summaries may be particularly useful for policymakers without a strong health or research background. Although our respondents had a higher-than-expected level of healthcare and research experience, their responses clearly indicated that all proposed components of the summaries of evidence have practical applications in health system decision processes. Comments strongly favoured clear, concise summaries in simple language, suggesting that researchers should consider ease of interpretation by policymakers with limited scientific literacy when preparing summaries.

The strengths of this survey were a good response rate from a diverse range of countries, backgrounds, levels of decision-making, and organisations. The fact that 25% of the surveys were partially completed may have been due to survey length. The comparatively lower response rate from SURE partner countries means results are likely biased towards higher-resource settings, limiting their applicability to resource-constrained settings. One significant limitation of the survey was the relatively high level of healthcare and research experience amongst respondents; only four respondents had no healthcare or research experience. This is probably not representative of health policymakers in general and potentially limits the generalizability of our findings. Nonetheless, the fact that the criteria included in the DECIDE framework were regarded as important for decision-making by these respondents provides further support for their inclusion in a framework for going from evidence to health system decisions.

This survey confirmed the relevance of the criteria that we had identified and incorporated in the DECIDE framework for health system decisions and suggests that the framework is likely to be helpful for informing health system decisions. Further development and evaluation of the framework will be based on practical applications of the framework to health system and population health decisions and user testing [20]. Facets of the framework that will be addressed by user testing were adapted from the work by Morville et al. [21] and Rosenbaum et al. [22], and include 1) findability: can users locate what they are looking for?; 2) usefulness: does the framework have practical value for the user?; 3) usability: how easy and satisfying is the framework to use?; 4) understandability: do users understand the framework and the content correctly?; 5) credibility: is this framework/content trustworthy?; 6) desirability: is the framework something the user wants/responds positively to?; and 7) identification: does the framework feel like it was designed for “someone like me (the user)”? Further work will address the advantages and disadvantages of using the same *versus* different systems for grading evidence, further clarification of the included criteria, the need for additional criteria, and the perceptions of policymakers and stakeholders who do not have a research or health professional background.

## Conclusions

Health system decision-making requires careful consideration of a multitude of variables, such as the magnitude of the problem, the size of benefits and adverse effects, feasibility and acceptability, as well as resource and equity implications. Surveyed individuals supported the use of systems to grade the quality of evidence for health system decisions, but there was disagreement as to whether uniform or different grading systems should be used for health system and clinical decisions. Communication of evidence to policymakers and stakeholders involved in health system decisions should employ succinct summaries of measures of effect and the quality of evidence in clear and simple language.

## Abbreviations

DALY: Disability-adjusted life years; DECIDE: Developing and evaluating communication strategies to support informed decisions and practice based on evidence; GRADE: Grading of recommendations assessment development and evaluation; HRP: Special programme of research development and research training in human reproduction; NNT: Number needed to treat; SUPPORT: Supporting policy relevant reviews and trials; SURE: Supporting the use of research evidence for policy in African health systems; UNDP: United nations development programme; UNFPA: United nations population fund; WHO: World health organization.

## Competing interests

The authors declare that they have no competing interests.

## Author’s contributions

All the named authors contributed to the survey design and content. JPV and ADO administered the survey and JPV conducted the analysis. JPV and ADO prepared the initial draft of the manuscript and all authors contributed to and approved the final version of the manuscript. This manuscript represents the views of the named authors alone. All authors read and approved the final manuscript.

## Supplementary Material

Additional file 1: Table S1 Participant responses on quality of evidence and components of evidence summaries, stratified by research experience and country of origin. Differences in responses by respondent research experience and country of origin were tested using χ^2^ tests; none were significant at the *P* <0.05 level. Click here for file
