# Supplementary material for: Policymakers’ and other stakeholders’ perceptions of key considerations for health system decisions and the presentation of evidence to inform those considerations: an international survey
Source: Health Res Policy Syst. 2013 May 24;11:19. doi: 10.1186/1478-4505-11-19 (PMC3672010; doi:10.1186/1478-4505-11-19)
Supplement: Additional file 1: Table S1 — Participant responses on quality of evidence and components of evidence summaries, stratified by research experience and country of origin. Differences in responses by respondent research experience and country of origin were tested using χ2 tests; none were significant at the P <0.05 level. [file 1478-4505-11-19-S1.doc]

**Additional file 1. Survey**

**Supplementary Table 1** – Participant responses on quality of evidence and components of evidence summaries, stratified by research experience and country of origin.

|  | **Survey participants**  **N (%)** | **Respondent research experience** | | **Country of origin** | |
| --- | --- | --- | --- | --- | --- |
|  |  | **Had some previous research experience** | **Had no previous research experience** | **Developing countries**  **(Referred from SURE network)** | **Developed countries (referred from DECIDE network)** |
| **TOTAL** | **112** | **66** | **18** | **23** | **84** |
| **Systematic consideration of available evidence helps improve the decision-making process**  ***Respondents to this question***  Yes  Probably  Not sure  Probably not  No | ***87***  71 (81.6)  15 (17.2)  1 (1.2)  0 (0.0)  0 (0.0) | ***66***  55 (83.3)  10 (15.2)  1 (1.5)  0 (0.0)  0 (0.0) | ***18***  15 (83.3)  3 (16.7)  0 (0.0)  0 (0.0)  0 (0.0) | ***23***  11 (47.8)  4 (17.4)  1 (4.3)  0 (0.0)  0 (0.0) | ***84***  56 (66.7)  11 (13.1)  0 (0.0)  0 (0.0)  0 (0.0) |
| **A system of grading the quality of evidence can help improve health system decision-making processes.**  ***Respondents to this question***  Yes  Probably  Not sure  Probably not  No | ***87***  52 (59.7)  29 (33.3)  5 (5.8)  1 (1.2)  0 (0.0) | ***66***  41 (62.1)  19(28.8)  5 (7.6)  1 (1.5)  0 (0.0) | ***18***  10 (55.6)  8 (44.4)  0 (0.0)  0 (0.0)  0 (0.0) | ***23***  8 (34.8)  6 (26.1)  2 (8.7)  0 (0.0)  0 (0.0) | ***84***  42 (50.0)  21 (25.0)  3 (3.6)  1 (1.2)  0 (0.0) |
| **Do you think that a system for rating the quality of evidence should be consistent for different types of decisions or that there should be different systems for different types of decisions (e.g. for clinical decisions and for health system decisions)?**  ***Respondents to this question***  The SAME SYSTEM DEFINITELY should be used for rating the quality of evidence for clinical and health system decisions  The SAME SYSTEM PROBABLY should be used for rating the quality of evidence for clinical and health system decisions  Neutral  A DIFFERENT SYSTEM PROBABLY should be used for rating the quality of evidence for health system decisions than for clinical decisions  A DIFFERENT SYSTEM DEFINITELY should be used for rating the quality of evidence for health system decisions than for clinical decisions | ***86***  9 (10.5)  24 (27.9)  14 (16.3)  27 (31.4)  12 (13.9) | ***66***  7 (10.6)  20 (30.3)  9 (13.6)  19 (28.8)  11 (16.7) | ***18***  2 (11.1)  4 (22.2)  5 (27.8)  6 (33.3)  1 (5.6) | ***23***  1 (4.3)  2 (8.7)  3 (13.0)  6 (26.1)  4 (17.4) | ***84***  7 (8.3)  22 (26.2)  11 (13.1)  20 (23.8)  6 (7.1) |
| **How important would you say it is to include a description of the size of effect in a summary of findings of research about the effects of a health systems intervention?**  ***Respondents to this question***  Important  Probably important  Not sure  Probably not important  Not important  I don’t know what that is | ***85***  64 (75.3)  17 (20.0)  2 (2.4)  2 (2.4)  0 (0.0)  0 (0.0) | ***66***  52 (78.8)  10 (15.2)  2 (3.0)  2 (3.0)  0 (0.0)  0 (0.0) | ***18***  11 (61.1)  7 (38.9)  0 (0.0)  0 (0.0)  0 (0.0)  0 (0.0) | ***15***  11 (73.3)  3 (20.0)  1 (6.7)  0 (0.0)  0 (0.0)  0 (0.0) | ***66***  49 (74.2)  14 (21.2)  1 (1.5)  2 (3.0)  0 (0.0)  0 (0.0) |
| **How important would you say it is to include confidence intervals for estimates of effects in a summary of findings of research about the effects of a health systems intervention?**  ***Respondents to this question***  Important  Probably important  Not sure  Probably not important  Not important  I don’t know what that is | ***85***  34 (40.0)  29 (34.1)  6 (7.1)  11 (12.9)  4 (4.7)  1 (1.2) | ***66***  29 (43.9)  21 (31.8)  4 (6.1)  9 (13.6)  3 (4.5)  0 (0.0) | ***18***  5 (27.8)  8 (44.4)  1 (5.6)  2 (11.1)  1 (5.6)  1 (5.6) | ***15***  7 (46.7)  3 (20.0)  1 (6.7)  3 (20.0)  1 (6.7)  0 (0.0) | ***66***  25 (37.9)  25 (37.9)  5 (7.6)  7 (10.6)  3 (4.5)  1 (1.5) |
| **How important would you say it is to include a description of the size of the effect in words in a summary of findings of research about the effects of a health systems intervention?**  ***Respondents to this question***  Important  Probably important  Not sure  Probably not important  Not important  I don’t know what that is | ***85***  48 (56.5)  27 (31.8)  7 (8.2)  2 (2.4)  1 (1.2)  0 (0.0) | ***66***  38 (57.6)  20 (30.3)  5 (7.6)  2 (3.0)  1 (1.5)  0 (0.0) | ***18***  9 (50.0)  7 (38.9)  2 (11.1)  0 (0.0)  0 (0.0)  0 (0.0) | ***15***  8 (53.3)  4 (26.7)  1 (6.7)  2 (13.3)  0 (0.0)  0 (0.0) | ***66***  37 (56.1)  22 (33.3)  6 (9.1)  0 (0.0)  1 (1.5)  0 (0.0) |
| **How important would you say it is to include how much evidence (the number of studies and/or participants) is available for each estimate of effect in a summary of findings of research about the effects of a health systems intervention?**  ***Respondents to this question***  Important  Probably important  Not sure  Probably not important  Not important  I don’t know what that is | ***85***  39 (45.9)  28 (32.9)  9 (10.6)  8 (9.4)  1 (1.2)  0 (0.0) | ***66***  28 (42.4)  25 (37.9)  5 (7.6)  7 (10.6)  1 (1.5)  0 (0.0) | ***18***  11 (61.1)  2 (11.1)  4 (22.2)  1 (5.6)  0 (0.0)  0 (0.0) | ***15***  6 (40.0)  5 (33.3)  1 (6.7)  3 (20.0)  0 (0.0)  0 (0.0) | ***66***  30 (45.5)  23 (34.8)  8 (12.1)  4 (6.1)  1 (1.5)  0 (0.0) |
| **How important would you say it is to include the quality of the evidence (how confident we are for each estimate of effect) in a summary of findings of research about the effects of a health systems intervention?**  ***Respondents to this question***  Important  Probably important  Not sure  Probably not important  Not important  I don’t know what that is | ***85***  59 (69.4)  23 (27.1)  3 (3.5)  0 (0.0)  0 (0.0)  0 (0.0) | ***66***  48 (72.7)  16 (24.2)  2 (3.0)  0 (0.0)  0 (0.0)  0 (0.0) | ***18***  11 (61.1)  6 (33.3)  1 (5.6)  0 (0.0)  0 (0.0)  0 (0.0) | ***15***  7 (46.7)  7 (46.7)  1 (6.7)  0 (0.0)  0 (0.0)  0 (0.0) | ***66***  49 (74.2)  15 (22.7)  2 (3.0)  0 (0.0)  0 (0.0)  0 (0.0) |

Differences in responses by respondent research experience and country of origin were tested using chi-square tests; none were significant at the p<0.05 level.
